# Associations between various obesity-related indicators and hypertension: a national cross-sectional survey with 117,609 Chinese adults

**DOI:** 10.3389/fpubh.2026.1925175

**Published:** 2026-08-28

**Authors:** Chenglong Wang, Yini Wu, Mingjian Nie, Duobujie, Chenhui Xiao, Wanglong He, Chaoqun Fan, Qiang Feng, Jingjing Wang

**Affiliations:** 1 National Physical Fitness and Scientific Exercise Research Center, China Institute of Sport Science, Beijing, China; 2 School of Physical Education, Beijing Jiaotong University, Beijing, China; 3 Tibet Institute of Sport Science, Lhasa, China; 4 Key Laboratory of Plateau Sports and Health of XiZang Autonomous Region, Lhasa, China

**Keywords:** body roundness index, waist-to-height ratio, hypertension, Chinese adults, dose-response relationships

## Abstract

**Objective:**

To evaluate how well obesity-related indicators (ORIs) discriminate hypertension in Chinese adults, thereby identifying the best-performing metrics and clarifying their dose–response associations with hypertension.

**Methods:**

This study analyzed data from the 2020 National Physical Fitness Surveillance Database, comprising 117,609 adults aged 20–79 years with complete data on 10 ORIs and relevant covariates. Receiver Operating Characteristic (ROC) curves were employed to evaluate the discriminatory ability of ORIs for hypertension, with Youden’s Index (sensitivity + specificity −1) used to determine optimal cut-off values for identifying individuals at high risk. Based on the comparative evaluation, ORIs with the highest AUC values were selected for further analysis using multivariable logistic regression and restricted cubic spline (RCS) models to investigate their dose–response relationships with hypertension.

**Results:**

The weighted prevalence of hypertension among Chinese adults was 24.79%. Among the 10 ORIs, body roundness index (BRI) and waist-to-height ratio (WHtR) exhibited the highest discriminatory ability for hypertension in the overall population and across sex-stratified subgroups (AUC: overall = 0.686, males = 0.647, females = 0.721). The optimal cut-off values for BRI and WHtR in the overall population were 3.66 and 0.52, respectively. Multivariable logistic regression models demonstrated that each unit increase in BRI z-score (OR = 1.51, 95% CI: 1.48–1.53) and WHtR z-score (OR = 1.53, 95% CI: 1.50–1.55) was associated with a 51% increased possibility of hypertension in the overall population. Individuals with BRI and WHtR values at or above the identified thresholds had a 2.00-fold and 1.97-fold increased possibility of hypertension, respectively, compared with those below the thresholds. RCS revealed a nonlinear dose–response relationship between BRI/WHtR and hypertension (*p* for nonlinear <0.05). Sensitivity analyses substantiated the robustness of these discriminatory and associative findings.

**Conclusion:**

Both BRI and WHtR, as abdominal obesity indices that simultaneously incorporate waist circumference and height, demonstrated highly consistent performance in discriminating hypertension among adults. BRI values below 3.66 and WHtR values below 0.52 potentially conferring a substantial reduction in hypertension possibility.

## Graphical abstract

**Figure:**
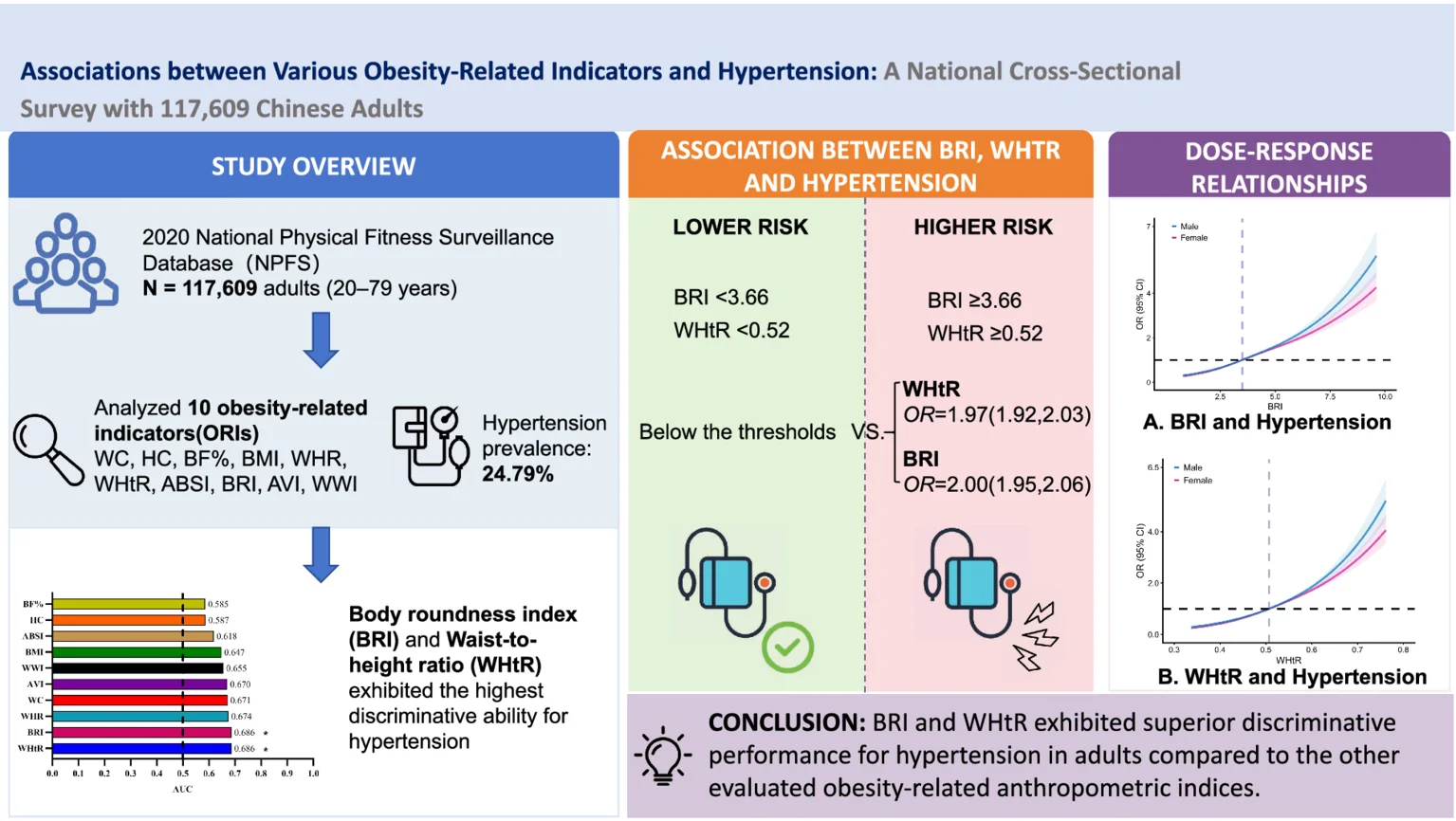

## 1 Introduction

Hypertension is a leading global chronic non-communicable disease, contributing significantly to morbidity and mortality (1). According to the 2023 China Cardiovascular Health and Disease Report, 31.6% of Chinese adults—approximately 245 million individuals—are affected by hypertension (2). As a major risk factor for stroke, coronary heart disease, chronic kidney disease, and other cardiovascular conditions, hypertension exerts a considerable burden on both individual health and socioeconomic systems (3). Early identification, risk stratification, and effective management of hypertension in adults are paramount. Obesity is a key determinant of blood pressure, and obesity-related indicators (ORIs) have been established as effective tools for discriminating hypertension due to their simplicity and practicality. These indicators provide a foundation for early personalized weight management, help to call for specific populations to adjust obesity-related indices through lifestyle modifications in order to achieve improved hypertension control (4, 5) and the formulation of evidence-based health promotion policies.

Existing evidence on the discriminatory ability of anthropometric indicators for hypertension remains limited and inconsistent across populations. Among the few available studies, findings vary significantly by ethnicity. For instance, waist-to-height ratio (WHtR) showed the strongest screening ability for hypertension in Iranian adults (AUC: males = 0.64, females = 0.67, 6), while a body shape index (ABSI) performed best in a United States cohort (AUC = 0.654) (6). Among Chinese populations, studies report divergent results. WHtR and body roundness index (BRI) demonstrated consistent discriminatory accuracy (AUC: overall = 0.665) (7), while other studies identified waist-to-hip ratio (WHR) (8) or Chinese Visceral Adiposity Index (CVAI) (9) as optimal indicators. These discrepancies may reflect methodological limitations, including small sample sizes and lack of nationally representative data—particularly in Chinese studies, where most studies are regional rather than population-wide. Further large-scale, ethnically balanced studies are needed to establish robust, generalizable thresholds.

Using nationally representative data from China, this study aimed to evaluate the discriminatory ability of anthropometric indicators for hypertension among Chinese adults and to investigate the associations between optimal ORIs and hypertension, with a specific focus on their dose–response relationships. These findings provide robust scientific evidence to inform weight management strategies and offer theoretical support for enhancing hypertension prevention policies.

## 2 Methods

### 2.1 Study design and participants

This study utilized data from the 2020 National Physical Fitness Surveillance (NPFS), the updated wave of national survey. This survey is led by the General Administration of Sport of China, designed to systematically monitor and evaluate trends and characteristics of physical fitness among the Chinese population (10). The NPFS employs a multistage, stratified probability cluster sampling design to ensure national representativeness, with standardized testing conducted across 31 provinces, autonomous regions, and municipalities in mainland China. Detailed sampling procedures have been previously described (11). After excluding individuals with missing data on blood pressure or anthropometric measurements, 117,609 adults were included in the final analysis. The study design and protocol were approved by the Ethics Review Committee of the China Institute of Sport Science (CISSLA-20240219). Written informed consent was obtained from all participants at enrolment.

### 2.2 Definition of hypertension

The diagnosis of hypertension was based on the criteria recommended by Chinese guidelines for the management of hypertension (12), defined as systolic blood pressure (SBP) ≥ 140 mmHg and/or diastolic blood pressure (DBP) ≥ 90 mmHg, or the patient were taking antihypertensive medication.

### 2.3 Anthropometric measurements and ORIs

Anthropometric measurements were performed by trained technicians following standardized protocols, as described previously (11, 13). All measurements were conducted with participants standing upright, wearing light clothing, and without shoes. Height was measured using a calibrated stadiometer (Jianmin, GMCS-SGJ3, China), and weight was measured using a calibrated digital scale (Jianmin, GMCS-RCS3, China). Waist circumference (WC) and hip circumference (HC) were measured using an anthropometric tape (Jianmin, GMCS-WD3, China). For WC, the tape was placed horizontally at the midpoint between the lower margin of the rib cage and the upper margin of the iliac crest in the mid-axillary line, measured at the end of normal exhalation, ensuring the tape was snug against the skin without compression. HC was measured at the level of maximum hip protrusion in the horizontal plane, with the tape placed horizontally around the hips. Body fat percentage (BF%) was determined using a bioimpedance body composition analyzer (Jianmin, GMCS-TZL3, China). Participants stood barefoot on the analyzer, free of metal objects, with hands and feet in contact with eight electrodes (two on the palms, two on the thumbs, two on the toes, and two on the heels).

The common 10 ORIs were included: waist circumference (WC), hip circumference (HC), body fat percentage (BF%), body mass index (BMI) (14), waist-to-hip ratio (WHR) (15), waist-to-height ratio (WHtR) (16), a body shape index (ABSI) (6), body roundness index (BRI) (17), abdominal volume index (AVI) (8), and weight-adjusted waist index (WWI) (18). Detailed formulas of ORIs are provided in the Supplementary materials.

### 2.4 Covariate

This study incorporated multiple covariates, including demographic information and lifestyle factors (9, 17, 19). Demographic characteristics comprised age, sex (male, female), residence (urban, rural), occupation (employed, unemployed, retired), education level (below high school, high school level, above high school education), and marital status (married, unmarried, widowed/divorced/separated). Lifestyle factors included smoking status and alcohol consumption (never, current) and self-reported regular exercise behavior (yes, no), which was defined as participating in physical exercise at least once a week in the past 6 months. Health status indicators encompassed the prevalence of hyperlipidemia and diabetes.

### 2.5 Statistical analysis

Statistical analyses were conducted using SPSS software (version 27.0). The normality of continuous variables was assessed with the Kolmogorov–Smirnov test. Normally distributed continuous variables were reported as means ± standard deviations, with comparisons between two groups performed using independent samples *t*-tests and among multiple groups using analysis of variance (ANOVA). Categorical variables were expressed as counts and percentages, with intergroup comparisons conducted using the χ^2^ test. Hypertension prevalence was weighted to account for the complex sampling design and stratification by sex and age groups. Post-stratification weights were calculated based on provincial regions, sex and age groups, using the 2020 Chinese census data as the reference population.

The discriminatory performance of ORIs for identifying hypertension in adults was assessed using the area under the curve (AUC) of receiver operating characteristic (ROC) curves. Optimal cut-off values for ORIs were determined by maximizing Youden’s index (sensitivity + specificity −1). The DeLong test was used to compare AUCs and identify indicators with superior discriminatory ability.

Multivariable logistic regression models were employed to assess the associations between ORIs and hypertension, with ORIs analyzed in two forms: (1) as continuous variables (*z*-scores: [observation − mean]/SD) and (2) as dichotomous variables (above vs. below established cut-off values). All models were adjusted for covariates, including demographic characteristics, lifestyle factors, and health status. Restricted cubic spline (RCS) models were employed to investigate the dose–response relationships between anthropometric indicators with high discriminatory performance for hypertension, adjusted for relevant covariates. RCS plots were generated using the ggplot2 package in R (version 4.4.0). A sensitivity analysis was conducted by re-running all statistical analyses with a redefined hypertension threshold (SBP ≥ 135 mmHg and/or DBP ≥ 85 mmHg). A 2-sided *p*-value <0.05 significance threshold was used to determine statistically significant differences.

## 3 Results

### 3.1 Characteristics of the participants

A total of 117,609 adults were included in the study. The mean age was 46.95 ± 16.40 years, with males accounting for 49.4% (58,042/117,609) and females 50.6% (59,567/117,609). The weighted prevalence of hypertension was 24.79% (95% CI: 23.90–25.68%). For the overall population, the mean values of ORIs were as follows: HC 95.19 ± 6.73 cm, WC 83.83 ± 10.18 cm, BF% 26.57 ± 6.91%, BMI 24.27 ± 3.35 kg/m^2^, WHR 0.88 ± 0.08, WHtR 0.52 ± 0.06, ABSI 0.08 ± 0.01, BRI 3.63 ± 1.23, AVI 14.39 ± 3.35, and WWI 10.45 ± 0.88. Compared with non-hypertension group, hypertension group exhibited significantly higher values for all ORIs (*p* < 0.05). Detailed results are presented in Table 1.

**Table 1:** Participants characteristic.

Characteristic	Overall (*n* = 117,609)	Non-hypertension (*n* = 77,796)	Hypertension (*n* = 39,813)	*p*
Height (cm)	162.82 ± 8.54	163.10 ± 8.42	162.28 ± 8.73	<0.01
Weight (kg)	64.51 ± 11.19	63.21 ± 10.95	67.05 ± 11.23	<0.01
WC (cm)	83.83 ± 10.18	81.79 ± 9.95	87.83 ± 9.41	<0.01
HC (cm)	95.19 ± 6.73	94.51 ± 6.62	96.54 ± 6.72	<0.01
BF% (%)	26.57 ± 6.91	25.85 ± 6.85	27.98 ± 6.80	<0.01
BMI (kg/m^2^)	24.27 ± 3.35	23.69 ± 3.24	25.39 ± 3.28	<0.01
WHR	0.88 ± 0.08	0.86 ± 0.07	0.91 ± 0.07	<0.01
WHtR	0.52 ± 0.06	0.50 ± 0.06	0.54 ± 0.06	<0.01
ABSI	0.08 ± 0.01	0.08 ± 0.01	0.08 ± 0.01	<0.01
BRI	3.63 ± 1.23	3.47 ± 1.15	4.25 ± 1.22	<0.01
AVI	14.39 ± 3.35	13.72 ± 3.21	15.69 ± 3.24	<0.01
WWI	10.45 ± 0.88	10.32 ± 0.86	10.77 ± 0.85	<0.01
SBP (mmHg)	128.23 ± 16.89	119.68 ± 11.38	144.93 ± 12.93	<0.01
DBP (mmHg)	77.89 ± 11.55	73.07 ± 8.66	87.31 ± 10.64	<0.01
Age (years)	46.95 ± 16.40	42.62 ± 15.27	55.40 ± 15.19	<0.01
Sex (%)				<0.01
Male	58,042(49.4)	35,519 (45.7)	22,523 (56.6)	
Female	59,567 (50.6)	42,277 (54.3)	17,290 (43.4)	
Residence [*n* (%)]				
Rural	43,850 (37.3)	27,132 (34.9)	16,718 (42.0)	
Urban	73,759 (62.7)	50,664 (65.1)	23,095 (58.0)	
Employment [*n* (%)]				<0.01
Employed	80,302 (68.3)	59,127 (76.0)	21,175 (53.2)	
Unemployed	9,404 (8.0)	6,619 (8.5)	2,785 (7.0)	
Retired	27,903 (23.7)	12,050 (15.5)	15,853 (39.8)	
Education [*n* (%)]				<0.01
Below	45,187 (38.4)	24,478 (31.5)	20,709 (52.0)	
High school	45,862 (39.0)	32,212 (41.4)	13,650 (34.3)	
Above	26,560 (22.6)	21,106 (27.1)	5,454 (13.7)	
Marital status [*n* (%)]				<0.01
Married	93,633 (79.6)	59,557 (76.6)	34,076 (85.6)	
Never married	17,653 (15.0)	15,012 (19.3)	2,641 (6.6)	
Widowed/divorced/separated	6,323 (5.4)	3,227 (4.1)	3,096 (7.8)	
Smoke [*n* (%)]	29,982 (25.5)	18,639 (24.0)	11,343 (28.5)	<0.01
Alcohol [*n* (%)]	43,434 (36.9)	28,017 (36.0)	15,417 (38.7)	<0.01
Exercise [*n* (%)]	57,511 (48.9)	38,587 (49.6)	18,871 (47.4)	<0.01
Hyperlipidemia [*n* (%)]	35,691 (30.3)	19,843 (25.5)	15,848 (39.8)	<0.01
Diabetes [*n* (%)]	10,620 (9.0)	5,304 (6.8)	5,316 (13.3)	<0.01

WC, waist circumference; HC, hip circumference; BF%, body fat percentage; BMI, body mass index; WHR, waist-hip ratio; WHtR, waist- height ratio; ABSI, a body shape index; BRI, body roundness index; AVI, abdominal volume index; WWI, weight-adjusted-waist index; SBP, systolic blood pressure; DBP, diastolic blood pressure.

### 3.2 ROC analyses of ORIs for discriminating hypertension

ROC curves demonstrated that the AUC for all ORIs were statistically significant (*p* < 0.05). The DeLong test revealed that the BRI and WHtR exhibited the highest discriminatory ability among all ORIs in the overall population and sex-stratified subgroups (all p < 0.05) (Supplementary Tables S1–S3). The optimal cut-off values for discriminating hypertension in the overall population were 0.52 for WHtR and 3.66 for BRI. In males, the cut-off value was 0.52 for WHtR and 3.71 for BRI, while in females, they were 0.52 for WHtR and 3.66 for BRI (Table 2).

**Table 2:** ROC curve analysis results for ORIs discriminating hypertension in adults (*n* = 117,609).

ORIs	Overall			Male			Female		
	AUC (95% CI)	Youden’s index^a^	Cut-off	AUC (95% CI)	Youden’s index^a^	Cut-off	AUC (95% CI)	Youden’s index ^a^	Cut-off
WC	0.671 (0.668–0.675)^b^	0.251	83.05	0.624 (0.619–0.629)^b^	0.185	86.95	0.701 (0.696–0.705)^b^	0.301	80.65
HC	0.587 (0.584–0.591)^b^	0.129	95.75	0.558 (0.553–0.562)^b^	0.086	96.55	0.598 (0.593–0.603)^b^	0.143	96.85
BF%	0.585 (0.582–0.589)^b^	0.115	26.45	0.610 (0.605–0.615)^b^	0.161	23.15	0.688 (0.684–0.693)^b^	0.280	30.85
BMI	0.647 (0.644–0.651)^b^	0.219	24.20	0.614 (0.609–0.618)^b^	0.170	24.70	0.669 (0.664–0.674)^b^	0.251	23.80
WHR	0.674 (0.671–0.677)^b^	0.256	0.88	0.631 (0.626–0.635)^b^	0.197	0.90	0.700 (0.696–0.705)^b^	0.298	0.85
WHtR	0.686 (0.683–0.689)^b^	0.278	0.52	0.647 (0.642–0.651)^b^	0.219	0.52	0.721 (0.716–0.725)^b^	0.331	0.52
ABSI	0.618 (0.615–0.621)^b^	0.182	0.07	0.576 (0.572–0.581)^b^	0.118	0.08	0.643 (0.638–0.648)^b^	0.223	0.08
BRI	0.686 (0.683–0.689) ^b^	0.278	3.66	0.647 (0.642–0.651)^b^	0.219	3.71	0.721 (0.716–0.725)^b^	0.331	3.66
AVI	0.670 (0.667-0.673)^b^	0.249	14.06	0.623 (0.618–0.628)^b^	0.183	15.24	0.699 (0.694–0.703)^b^	0.298	13.26
WWI	0.655 (0.651–0.658)	0.233	10.51	0.621 (0.617–0.629)	0.182	10.47	0.693 (0.688–0.698)	0.295	10.51

a Cut-off values were determined using the maximum Youden’s Index.

b *p* < 0.05.

ORIs, Obesity-Related Indicators; WC, waist circumference; HC, hip circumference; BF%, body fat percentage; BMI, body mass index; WHR, waist-hip ratio; WHtR, waist- height ratio; ABSI, a body shape index; BRI, body roundness index; AVI, abdominal volume index; WWI, weight-adjusted-waist index; ROC, Receiver Operating Characteristic; AUC, Area Under the Curve.

### 3.3 The association of optimal ORIs and hypertension possibility

As presented in Table 3, each 1-SD increase in the BRI was associated with 51% increased odds of prevalent hypertension in the overall adult population (OR = 1.51, 95% CI: 1.48–1.53). Similarly, a 1-SD increase in the WHtR corresponded to a 53% elevated hypertension (OR = 1.53, 95% CI: 1.50–1.55). Sex-stratified regression analyses revealed consistent trends (all *p* < 0.05). Based on the cut-off values derived from ROC curves, BRI and WHtR were dichotomized into two groups (at or above threshold vs. below the threshold) for further regression analysis. Individuals with BRI and WHtR at or above the thresholds had a 2.00-fold (OR = 2.00, 95% CI: 1.95–2.06) and 1.97-fold (OR = 1.97, 95% CI: 1.92–2.03) higher odds of prevalent hypertension, respectively, compared to those below the thresholds.

**Table 3:** The association of BRI, WHtR and hypertension in multivariate logistic regression models.

ORIs	Overall	*p*	Male	*p*	Female	*p*
	OR (95% CI)		OR (95% CI)		OR (95% CI)	
BRI						
BRI *z*-score	1.51 (1.48, 1.53)	<0.01	1.49 (1.46, 1.53)	<0.01	1.44 (1.40, 1.46)	<0.01
Dichotomous category of BRI						
<3.66	Ref (1.00)		Ref (1.00)		Ref (1.00)	
≥3.66	2.00 (1.95, 2.06)	<0.01	1.87 (1.80, 1.94)	<0.01	1.88 (1.80, 1.97)	<0.01
WHtR						
WHtR *z*-score	1.53 (1.50, 1.55)	<0.01	1.50 (1.47, 1.54)		1.46 (1.43, 1.49)	<0.01
Dichotomous category of WHtR						
<0.52	Ref (1.00)		Ref (1.00)		Ref (1.00)	
≥0.52	1.97 (1.92, 2.03)	<0.01	1.86 (1.79, 1.92)	<0.01	1.87 (1.79, 1.96)	<0.01

The models were adjusted for the following covariates: age, residence, employment, education, marital status, smoking, alcohol status, exercise behavior, hyperlipidemia and diabetes.

### 3.4 Dose–response relationship between optimal ORIs and hypertension

**Figure 1:**
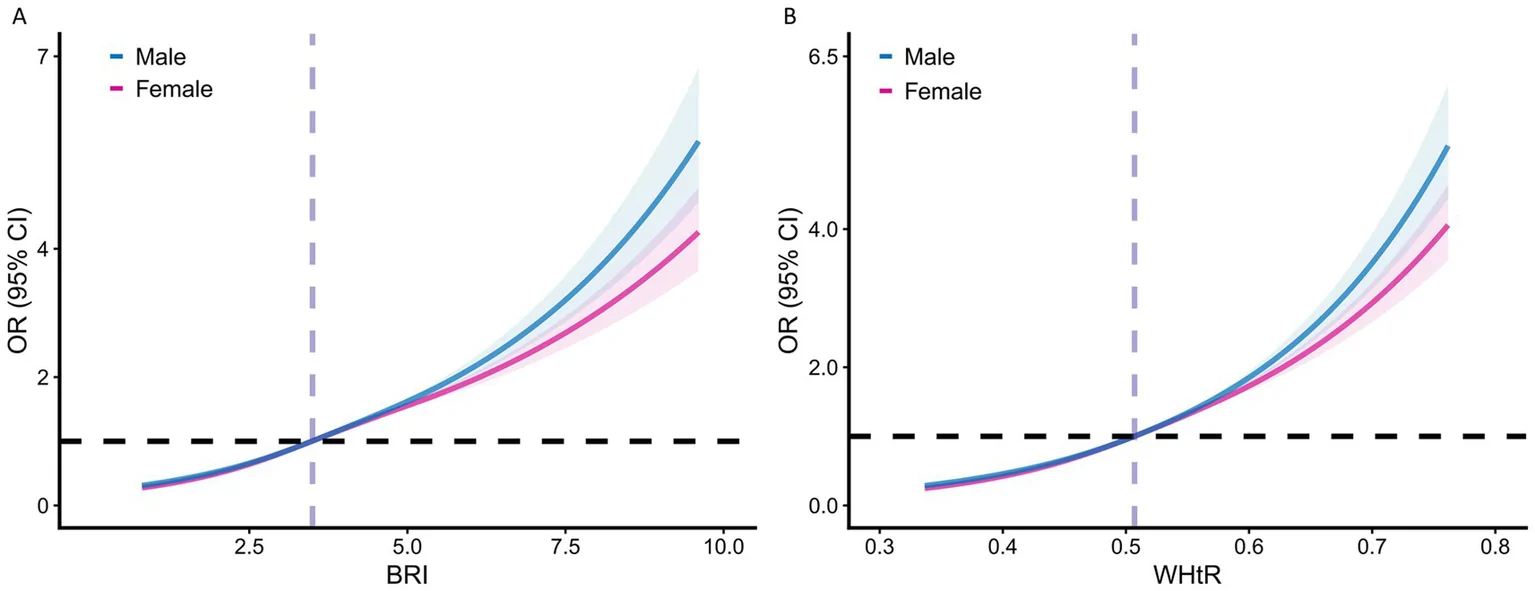
Dose–response relationship between BRI, WHtR, and hypertension. **(A)** Dose–response relationship between BRI and hypertension. **(B)** Dose–response relationship between WHtR and hypertension. The models were adjusted for the following covariates: age, residence, employment, education, marital status, smoking, alcohol status, exercise behavior, hyperlipidemia, and diabetes.

Figure 1 presents sex-stratified RCS models analyzing the dose–response relationships between BRI, WHtR, and hypertension in adults. After adjusting for covariates (age, residence, occupation, education, marital status, smoking, alcohol status, exercise behavior, hyperlipidemia and diabetes), both BRI and WHtR exhibited a gradually increasing trend in their association with hypertension (all *p* for nonlinear <0.05). The model further demonstrated a steep escalation in hypertension once BRI and WHtR surpassed their respective threshold values.

### 3.5 Sensitivity analysis

For sensitivity analysis, we adopted an alternative blood pressure criterion (SBP ≥ 135 mmHg and/or DBP ≥ 85 mmHg) as recommended by previous literature (8), We then reassessed the discriminatory ability of ORIs for hypertension and conducted regression analyses with adjustment for all covariates. The results confirmed that BRI and WHtR consistently demonstrated superior discriminatory performance among all included ORIs (AUC = 0.677), with similar findings observed across sex-stratified analyses (males: AUC = 0.637; females: AUC = 0.697) (Supplementary Tables S4–S11). In multivariable logistic regression models, each standard deviation increase in BRI was associated with a 52% increased possibility of hypertension in males (OR = 1.52, 95% CI: 1.49–1.56) and a 44% increased possibility in females (OR = 1.44, 95% CI: 1.41–1.47). Likewise, each standard deviation increase in WHtR corresponded to a 52% elevated possibility of hypertension in males (OR = 1.52, 95% CI: 1.49–1.55) and a 45% elevated possibility in females (OR = 1.45, 95% CI: 1.42–1.48) (Supplementary Table S12).

## 4 Discussion

This study, utilizing a nationally representative sample, conducted a comprehensive comparative analysis of ten obesity-related anthropometric indicators to evaluate their discriminatory capacity for hypertension in adults. We further investigated the associations between the highest-performing discriminatory indicators and hypertension possibility. Our findings demonstrated that BRI and WHtR consistently exhibited superior discriminatory performance across both the overall sample and sex-stratified analyses. Multivariable logistic regression models confirmed significant positive associations between BRI z-score, WHtR z-score, and hypertension. When analyzed using optimal cut-off values derived from ROC analyses, individuals at or above the established thresholds demonstrated a 2.00-fold and 1.97-fold higher possibility of hypertension, respectively, compared to those below the thresholds, with consistent patterns observed across sexes. RCS models revealed a gradually increasing dose–response relationship between both BRI and WHtR and hypertension possibility, suggesting non-linear associations at different exposure levels. Sensitivity analyses further corroborated the robustness of these discriminatory and associative findings. These findings may provide a valuable reference to the application of anthropometric indicators in cardiovascular health management. Results from the dose–response analysis imply that maintaining BRI and WHtR below critical thresholds could serve as a preventive strategy against hypertension.

Hypertension affects one in four Chinese adults with the weighted prevalence at 24.79%. This result consistent with the 2012–2015 China Hypertension Survey (weighted prevalence: 23.2%) and the 2018 China Chronic Disease and Risk Factor Surveillance (weighted prevalence: 27.5%) (20). Hypertension possibility is significantly associated with overweight and obesity prevalence. The application of obesity-related anthropometric indicators for early hypertension identification represents a practical approach to facilitate timely prevention, thereby reducing the burden of hypertension and its associated cardiovascular complications. This strategy aligns with proactive health risk management paradigms. This study used ROC analysis to compare the discriminatory performance of 10 obesity-related anthropometric indicators for hypertension in adults. The results indicated that BRI and WHtR exhibited the highest discriminatory ability for hypertension in the overall population, as well as in adult males and females, aligning with findings from Feng et al. (7), Wang et al. (21), and Huang Yali et al. (22). BRI, developed by Thomas et al. (23) in 2013, is a geometric model derived from anthropometric data across three databases, incorporating dual-energy X-ray absorptiometry for body fat mass and magnetic resonance imaging for visceral fat volume. Using regression models based on eccentricity and other variables, BRI approximates the human body shape as an ellipse and provides a formula to predict body fat percentage and visceral fat percentage. BRI serves as a visual tool for assessing body fat distribution and predicting overall health risks. WHtR, initially proposed concurrently by Japanese and British researchers as a method for assessing body shape and monitoring health risks, was later refined by Ashwell and Gibson (24), who established a critical threshold (<0.50) and promoted its use as an economical, simple, and rapid screening tool for health risks. The European Association for the Study of Obesity’s latest expert consensus also recognizes WHtR >0.50 as a criterion for clinical obesity diagnosis (16). Accounting for racial differences and outcome variables, the critical threshold of 0.52 identified in this study is close to the established standard. Notably, both BRI and WHtR share similarities in their calculation formulas, as both integrate two fundamental anthropometric dimensions: body circumference and height-adjusted measurements. This may also explain why they exhibit consistent efficacy in distinguishing hypertension. Therefore, our findings may further emphasize that height-normalized central obesity indices may demonstrate a more robust pathophysiological association with hypertension development in adults. The physiological mechanisms underlying the association between central obesity and hypertension likely involve excessive abdominal adiposity accumulation, which precipitates increased insulin resistance. This insulin resistance may synergistically modulate the obesity-hypertension pathway through enhanced adipokine secretion and augmented sympathetic nervous system activity (25). Furthermore, insulin resistance has been demonstrated to induce and potentiate the renin-angiotensin-aldosterone system (26), promote vascular endothelial dysfunction (27), and elevate peripheral vascular resistance (28), thereby contributing to blood pressure dysregulation and subsequent hypertension development.

This investigation further examined the two high-efficacy discriminatory indicators, BRI and WHtR, to elucidate their associations with hypertension in Chinese adults. Multivariable logistic regression analyses revealed that incremental elevations in BRI and WHtR values were significantly associated with an augmented odd of prevalent hypertension. Adults whose BRI equaled or exceeded the identified critical threshold (3.66) and whose WHtR equaled or exceeded the critical threshold (0.52) demonstrated a substantially heightened odds of prevalent hypertension. Restricted cubic spline (RCS) analyses further delineated a nonlinear dose–response relationship between BRI, WHtR, and hypertension, which suggests a complex physiological interrelationship beyond simple linear correlation.

The literature reveals a paucity of studies directly investigating the dose–response relationship between BRI and hypertension. Nevertheless, a noteworthy investigation examining the association between BRI and incident hyperuricemia documented a comparable nonlinear relationship (*p* for nonlinear <0.01) (29). Zheng et al. (30) elucidated the combined effect of triglyceride-glucose index (TyG) and obesity indicators on hypertension possibility, demonstrating a near-linear trend when TyG was independently associated with hypertension, but a pronounced “J-curve” nonlinear trend when TyG was combined with WHtR. Regarding WHtR, Wang et al. (21) identified a nonlinear association between WHtR and incident hypertension possibility, corroborating our findings. Several investigations suggest that malnutrition (31) constitutes an independent health risk, and the adverse effects of low WHtR on hypertension may be attributed to frailty or nutritional deficiencies, although the precise underlying mechanisms remain incompletely characterized (32). Synthesizing existing evidence with the findings of our investigation, we hypothesize the existence of an optimal range for visceral adiposity in relation to hypertension prevalence in adults. At lower levels of BRI and WHtR, hypertension possibility increases gradually with rising indicator values, potentially reflecting physiological tolerance or compensatory regulatory mechanisms that attenuate the adverse effects of moderate visceral fat accumulation (33, 34). However, when BRI and WHtR exceed a certain threshold, hypertension possibility rises sharply. This may be driven by excessive visceral fat accumulation leading to insulin resistance (35) and the release of pro-inflammatory factors such as leptin (36), which accelerate insulin resistance and peripheral vascular resistance. These risk factors may create a “synergistic effect,” substantially increasing hypertension possibility. These findings underscore the critical importance of weight control, particularly early management of central obesity, to prevent metabolic disorders and the rapid escalation of hypertension.

This study utilized the most recent nationally representative data from the sports sector in China, employing rigorous statistical methods to compare and identify anthropometric indicators with optimal discriminatory efficacy for hypertension, thereby providing insights into their associations with hypertension for weight management, early identification, and prevention of hypertension. However, certain limitations should be acknowledged. As a cross-sectional study, it cannot establish causal relationships between BRI, WHtR, and hypertension in adults. Although a comprehensive set of obesity-related anthropometric indicators was included, some relevant indicators may have been overlooked. Prevalence estimates in this study were weighted according to the sampling design to yield nationally representative data; however, sampling weights were not incorporated into association analyses. Although key stratification variables, age, sex, and region, were included as covariates in the regression models, which largely adjusted for potential bias introduced by the sampling framework, we acknowledge that this analytical approach may compromise the generalizability of the absolute effect estimates. Future studies primarily aimed at developing national identifying tools should consider incorporating full sampling weights into regression analyses. The study found that while BRI and WHtR exhibited the highest AUC values among all assessed anthropometric indicators, they should not be interpreted as having strong or excellent discriminatory power for individual-level hypertension screening. Therefore, our findings should be interpreted with caution, and the clinical application of these cut-off values warrants further validation in external cohorts, preferably with the incorporation of additional metabolic or biochemical markers to improve discriminatory accuracy. Additionally, although the study adjusted for basic demographic information, lifestyle factors, and health conditions, it is important to consider potential confounding factors such as genetics, environmental conditions, and dietary salt intake, which were not fully accounted for in our analysis. Further studies should incorporate these variables into analytical models to explore the complex relationships between ORIs and hypertension, and well-designed cohort studies are warranted to validate our findings.

## 5 Conclusion

BRI and WHtR exhibited comparable and superior discriminative performance for hypertension in adults compared to the other evaluated obesity-related anthropometric indices. The research indicates that maintaining BRI < 3.66 and WHtR < 0.52 is significantly associated with reduced hypertension possibility. Notably, the observed dose–response relationships between these indices and hypertension suggests optimal risk reduction occurs when values remain within these threshold ranges.

These findings emphasize the clinical importance of population-specific weight management strategies and targeted primary healthcare interventions. Implementation of these more sensitive anthropometric measures could enhance early identification of at-risk individuals and facilitate more effective preventive approaches in clinical practice.

## Data Availability

The data used in this study are derived from 2020 National Physical Fitness Surveillance. Access to these data is restricted and requires formal application to the data owner, the General Administration of Sport of China (https://www.sport.gov.cn/n315/n329/). Due to the sensitive nature of the data and the potential for participant identification, the authors are unable to directly share the dataset with other researchers. However, inquiries regarding the data or potential collaborative research projects involving overlapping data are welcome and can be directed to Jingjing Wang (wangjingjing@ciss.cn).
