# Correction: Associations between various obesity-related indicators and hypertension: a national cross-sectional survey with 117,609 Chinese adults

**DOI:** 10.3389/fpubh.2026.1985987

**Published:** 2026-09-30

**Authors:** Chenglong Wang, Yini Wu, Mingjian Nie, Duobujie, Chenhui Xiao, Wanglong He, Chaoqun Fan, Qiang Feng, Jingjing Wang

**Affiliations:** 1 National Physical Fitness and Scientific Exercise Research Center, China Institute of Sport Science, Beijing, China; 2 School of Physical Education, Beijing Jiaotong University, Beijing, China; 3 Tibet Institute of Sport Science, Lhasa, China; 4 Key Laboratory of Plateau Sports and Health of XiZang Autonomous Region, Lhasa, China

**Keywords:** body roundness index, waist-to-height ratio, hypertension, Chinese adults, dose-response relationships

There was a mistake in Tables 1, 2 and 3 as published. Some values were incorrectly recorded.

**Table:** 

**Location**		**Current (incorrect)**
Table 1	Hypertension SBP	114.93 ± 12.93
Table 2	AVI Confidence Interval	0.670 (0.651–0.658)
Table 3	WHtR (female) Confidence Interval	1.87 (1.79, 1.86)

The corrected Tables 1, 2 and 3 appear below.

**Table 1:** Participants characteristic.

Characteristic	Overall (*n* = 117,609)	Non-hypertension (*n* = 77,796)	Hypertension (*n* = 39,813)	*P*
Height (cm)	162.82 ± 8.54	163.10 ± 8.42	162.28 ± 8.73	< 0.01
Weight (kg)	64.51 ± 11.19	63.21 ± 10.95	67.05 ± 11.23	< 0.01
WC (cm)	83.83 ± 10.18	81.79 ± 9.95	87.83 ± 9.41	< 0.01
HC (cm)	95.19 ± 6.73	94.51 ± 6.62	96.54 ± 6.72	< 0.01
BF% (%)	26.57 ± 6.91	25.85 ± 6.85	27.98 ± 6.80	< 0.01
BMI (kg/m^2^)	24.27 ± 3.35	23.69 ± 3.24	25.39 ± 3.28	< 0.01
WHR	0.88 ± 0.08	0.86 ± 0.07	0.91 ± 0.07	< 0.01
WHtR	0.52 ± 0.06	0.50 ± 0.06	0.54 ± 0.06	< 0.01
ABSI	0.08 ± 0.01	0.08 ± 0.01	0.08 ± 0.01	< 0.01
BRI	3.63 ± 1.23	3.47 ± 1.15	4.25 ± 1.22	< 0.01
AVI	14.39 ± 3.35	13.72 ± 3.21	15.69 ± 3.24	< 0.01
WWI	10.45 ± 0.88	10.32 ± 0.86	10.77 ± 0.85	< 0.01
SBP (mmHg)	128.23 ± 16.89	119.68 ± 11.38	144.93 ± 12.93	< 0.01
DBP (mmHg)	77.89 ± 11.55	73.07 ± 8.66	87.31 ± 10.64	< 0.01
Age (years)	46.95 ± 16.40	42.62 ± 15.27	55.40 ± 15.19	< 0.01
Sex (%)				< 0.01
Male	58,042 (49.4)	35,519 (45.7)	22,523 (56.6)	
Female	59,567 (50.6)	42,277 (54.3)	17,290 (43.4)	
Residence [*n* (%)]				
Rural	43,850 (37.3)	27,132 (34.9)	16,718 (42.0)	
Urban	73,759 (62.7)	50,664 (65.1)	23,095 (58.0)	
Employment [*n* (%)]				< 0.01
Employed	80,302 (68.3)	59,127 (76.0)	21,175 (53.2)	
Unemployed	9,404 (8.0)	6,619 (8.5)	2,785 (7.0)	
Retired	27,903 (23.7)	12,050 (15.5)	15,853 (39.8)	
Education [*n* (%)]				< 0.01
Below	45,187 (38.4)	24,478 (31.5)	20,709 (52.0)	
High school	45,862 (39.0)	32,212 (41.4)	13,650 (34.3)	
Above	26,560 (22.6)	21,106 (27.1)	5,454 (13.7)	
Marital status [*n* (%)]				< 0.01
Married	93,633 (79.6)	59,557 (76.6)	34,076 (85.6)	
Never married	17,653 (15.0)	15,012 (19.3)	2,641 (6.6)	
Widowed/divorced/separated	6,323 (5.4)	3,227 (4.1)	3,096 (7.8)	
Smoke [*n* (%)]	29,982 (25.5)	18,639 (24.0)	11,343 (28.5)	< 0.01
Alcohol [*n* (%)]	43,434 (36.9)	28,017 (36.0)	15,417 (38.7)	< 0.01
Exercise [*n* (%)]	57,511 (48.9)	38,587 (49.6)	18,871 (47.4)	< 0.01
Hyperlipidemia [*n* (%)]	35,691 (30.3)	19,843 (25.5)	15,848 (39.8)	< 0.01
Diabetes [*n* (%)]	10,620 (9.0)	5,304 (6.8)	5,316 (13.3)	< 0.01

WC, waist circumference; HC, hip circumference; BF%, body fat percentage; BMI, body mass index; WHR, waist-hip ratio; WHtR, waist- height ratio; ABSI, a body shape index; BRI, body roundness index; AVI, abdominal volume index; WWI, weight-adjusted-waist index; SBP, systolic blood pressure; DBP, diastolic blood pressure.

**Table 2:** ROC curve analysis results for ORIs discriminating hypertension in adults (*n* = 117,609).

ORIs	Overall			Male			Female		
	AUC (95%CI)	Youden's index^a^	Cut-off	AUC (95%CI)	Youden's index^a^	Cut-off	AUC (95%CI)	Youden's index^a^	Cut-off
WC	0.671 (0.668–0.675)^b^	0.251	83.05	0.624 (0.619–0.629)^b^	0.185	86.95	0.701 (0.696–0.705)^b^	0.301	80.65
HC	0.587 (0.584–0.591)^b^	0.129	95.75	0.558 (0.553–0.562)^b^	0.086	96.55	0.598 (0.593–0.603)^b^	0.143	96.85
BF%	0.585 (0.582–0.589)^b^	0.115	26.45	0.610 (0.605–0.615)^b^	0.161	23.15	0.688 (0.684–0.693)^b^	0.280	30.85
BMI	0.647 (0.644–0.651)^b^	0.219	24.20	0.614 (0.609–0.618)^b^	0.170	24.70	0.669 (0.664–0.674)^b^	0.251	23.80
WHR	0.674 (0.671–0.677)^b^	0.256	0.88	0.631 (0.626–0.635)^b^	0.197	0.90	0.700 (0.696–0.705)^b^	0.298	0.85
WHtR	0.686 (0.683–0.689)^b^	0.278	0.52	0.647 (0.642–0.651)^b^	0.219	0.52	0.721 (0.716–0.725)^b^	0.331	0.52
ABSI	0.618 (0.615–0.621)^b^	0.182	0.07	0.576 (0.572–0.581)^b^	0.118	0.08	0.643 (0.638–0.648)^b^	0.223	0.08
BRI	0.686 (0.683–0.689)^b^	0.278	3.66	0.647 (0.642–0.651)^b^	0.219	3.71	0.721 (0.716–0.725)^b^	0.331	3.66
AVI	0.670 (0.667–0.673)^b^	0.249	14.06	0.623 (0.618–0.628)^b^	0.183	15.24	0.699 (0.694–0.703)^b^	0.298	13.26
WWI	0.655 (0.651–0.658)	0.233	10.51	0.621 (0.617–0.629)	0.182	10.47	0.693 (0.688–0.698)	0.295	10.51

^a^Cut-off values were determined using the maximum Youden's Index.

^b^*p* < 0.05.

ORIs, Obesity-Related Indicators; WC, waist circumference; HC, hip circumference; BF%, body fat percentage; BMI, body mass index; WHR, waist-hip ratio; WHtR, waist- height ratio; ABSI, a body shape index; BRI, body roundness index; AVI, abdominal volume index; WWI, weight-adjusted-waist index; ROC, Receiver Operating Characteristic; AUC, Area Under the Curve.

**Table 3:** The association of BRI, WHtR and hypertension in multivariate logistic regression models.

ORIs	Overall	*P*	Male	*P*	Female	*P*
	*OR* (95%*CI*)		*OR* (95%*CI*)		*OR* (95%*CI*)	
BRI						
BRI z-score	1.51 (1.48, 1.53)	< 0.01	1.49 (1.46, 1.53)	< 0.01	1.44 (1.40, 1.46)	< 0.01
Dichotomous category of BRI						
< 3.66	Ref (1.00)		Ref (1.00)		Ref (1.00)	
≥3.66	2.00 (1.95, 2.06)	< 0.01	1.87 (1.80, 1.94)	< 0.01	1.88 (1.80, 1.97)	< 0.01
WHtR						
WHtR z-score	1.53 (1.50, 1.55)	< 0.01	1.50 (1.47, 1.54)		1.46 (1.43, 1.49)	< 0.01
Dichotomous category of WHtR						
< 0.52	Ref (1.00)		Ref (1.00)		Ref (1.00)	
≥0.52	1.97 (1.92, 2.03)	< 0.01	1.86 (1.79, 1.92)	< 0.01	1.87 (1.79, 1.96)	< 0.01

The models were adjusted for the following covariates: age, residence, employment, education, marital status, smoking, alcohol status, exercise behavior, hyperlipidemia and diabetes.

The original version of this article has been updated.

