# Supplementary material for: Associations between various obesity-related indicators and hypertension: a national cross-sectional survey with 117,609 Chinese adults
Source: Front Public Health. 2026 Aug 28;14:1925175. doi: 10.3389/fpubh.2026.1925175 (PMC13562001; doi:10.3389/fpubh.2026.1925175)
Supplement: Supplementary file 1 [file Data_Sheet_1.PDF]

Supplementary Material

1 Supplementary Data

Formulas of obesity-related indicators

$BMI = weight(kg)/height^2(m)$

$WHR = WC(cm)/HC(cm)$

$WHtR = WC(cm)/height(m)$

$ABSI = WC(cm)/(BMI^{\frac{2}{3}} \times height(m)^{\frac{1}{2}})$

$BRI = 364.2 - 365.5 \times \sqrt{[1\text{-}(\text{WC}(cm)/(\pi\times\text{height}(cm)))^2]}$

$AVI = [2 \times WC(cm)^2 + 0.7 \times (WC(cm) - HC(cm))^2]/1000$

$WWI = WC(cm)/\sqrt{weight(kg)}$

HC, hip circumference; WC, waist circumference

2 Supplementary Figures and Tables

Table S1. Comparisons of AUC for predicting hypertension among ORIs by the Delong test in overall

comparator	AUC diff (95%CI)	*Z*	*P*	*Pa*
WHtR - waist	-0.014(-0.016, -0.013)	-18.408	0.000	0.057
WHtR -hip	-0.099(-0.102, -0.096)	-64.166	0.000	0.058
WHtR -bodyfat	-0.101(-0.104, -0.097)	-61.184	0.000	0.058
WHtR - bmi	-0.039(-0.041, -0.036)	-30.782	0.000	0.057
WHR – WWI	0.019(0.017, 0.022)	14.906	0.000	0.057
WHtR - ABSI	0.068(0.065, 0.071)	44.632	0.000	0.057
**WHtR - BRI**	0.000(0.000, 0.000)	0.757	**0.449**	0.057
WHtR - AVI	0.016(0.014, 0.017)	19.965	0.000	0.057
WHtR - WWI	0.031(0.029, 0.033)	31.019	0.000	0.057
BRI - waist	-0.014(-0.016, -0.013)	-18.408	0.000	0.057

BRI - hip	-0.099(-0.102, -0.096)	-64.166	0.000	0.058
BRI - bodyfat	-0.101(-0.104, -0.097)	-61.184	0.000	0.058
BRI - WHR	-0.012(-0.014, -0.009)	-9.861	0.000	0.057
BRI - ABSI	-0.068(-0.071, -0.065)	-44.632	0.000	0.057
BRI - AVI	0.016(0.014, 0.017)	19.965	0.000	0.057
BRI - WWI	0.031(0.029, 0.033)	31.019	0.000	0.057
waist - hip	0.084(0.082, 0.87)	66.332	0.000	0.058
waist - bodyfat	0.086(0.082, 0.090)	44.444	0.000	0.058
waist – bmi	0.024(0.022, 0.03)	19.461	0.000	0.057
waist - WHR	-0.003(-0.005, -0.001)	-2.642	0.008	0.057
waist - ABSI	0.053(0.050, 0.056)	34.996	0.000	0.058
waist - AVI	0.001(0.001, 0.001)	32.879	0.000	0.057
waist - WWI	0.017(0.014, 0.019)	12.152	0.000	0.057
hip - bodyfat	0.002(-0.002, 0.006)	1.027	0.305	0.059
hip - bmi	-0.060(-0.063, -0.057)	-42.939	0.000	0.058
hip - WHR	-0.087(-0.091, -0.083)	-40.295	0.000	0.058
hip - ABSI	-0.031(-0.035, -0.026)	-14.144	0.000	0.059
hip - AVI	-0.083(-0.085, -0.080)	-66.929	0.000	0.058
hip - WWI	-0.068(-0.072, -0.064)	-33.061	0.000	0.058
bodyfat - bmi	-0.062(-0.065, -0.059)	-40.446	0.000	0.058
bodyfat - WHR	-0.089(-0.093, -0.085)	-40.334	0.000	0.058
bodyfat - ABSI	-0.033(-0.038, -0.028)	-13.221	0.000	0.059
bodyfat - AVI	-0.085(-0.089, -0.081)	-43.873	0.000	0.058
bodyfat - WWI	-0.069(-0.074, -0.065)	-33.469	0.000	0.058
bmi - WHR	-0.027(-0.030, -0.024)	-15.604	0.000	0.057
bmi - ABSI	0.029(0.025, 0.034)	12.090	0.000	0.058
bmi - BRI	-0.039(-0.041, -0.036)	-30.782	0.000	0.057
bmi - AVI	-0.023(-0.025, -0.020)	-18.489	0.000	0.057
bmi - WWI	-0.007(-0.012, -0.003)	-3.585	0.000	0.058
WHR - WHtR	-0.012(-0.014, -0.009)	-9.861	0.000	0.057

WHR - ABSI	0.056(0.054, 0.059)	40.614	0.000	0.058
WHR - AVI	0.004(0.002, 0.006)	3.789	0.000	0.057
ABSI - AVI	-0.052(-0.055, -0.049)	-33.782	0.000	0.058
ABSI - WWI	-0.037(-0.038, -0.035)	-45.800	0.000	0.058
AVI - WWI	0.015(0.013, 0.018)	11.048	0.000	0.057

Table S2. Comparisons of AUC for predicting hypertension among ORIs by the Delong test in male

**comparator**	**AUC diff (95%CI)**	***Z***	***P***	***Pa***
WHtR - waist	-0.023(-0.025, -0.021)	-24.320	0.000	0.068
WHtR - hip	-0.089(-0.093, -0.085)	-41.697	0.000	0.069
WHtR - bodyfat	-0.037(-0.041, -0.033)	-18.801	0.000	0.068
WHtR - bmi	-0.033(-0.037, -0.030)	-18.829	0.000	0.068
WHR - WHtR	-0.016(-0.019, -0.013)	-10.074	0.000	0.068
WHtR - ABSI	0.070(0.066, 0.075)	31.054	0.000	0.069
**WHtR - BRI**	0.000(0.000, 0.000)	1.541	**0.123**	0.068
WHtR - AVI	0.024(0.022, 0.026)	25.020	0.000	0.068
WHtR - WWI	0.025(0.022, 0.028)	16.826	0.000	0.068
BRI - waist	-0.023(-0.025, -0.021)	-24.320	0.000	0.068
BRI - hip	-0.089(-0.093, -0.085)	-41.697	0.000	0.069
BRI - bodyfat	-0.037(-0.041, -0.033)	-18.800	0.000	0.068
BRI - bmi	-0.033(-0.037, -0.030)	-18.829	0.000	0.068
WHR - BRI	-0.016(-0.019, -0.013)	-10.074	0.000	0.068
ABSI - BRI	-0.070(-0.075, -0.066)	-31.054	0.000	0.069
BRI - AVI	0.024(0.002, 0.026)	25.020	0.000	0.068
BRI - WWI	0.025(0.022, 0.028)	16.826	0.000	0.068
waist - hip	0.066(0.063, 0.070)	38.068	0.000	0.069
waist - bodyfat	0.014(0.011, 0.017)	7.893	0.000	0.069
waist – bmi	0.010(0.007, 0.014)	5.996	0.000	0.069

waist - WHR	-0.007(-0.010, -0.003)	-3.998	0.000	0.069
waist - ABSI	0.048(0.043, 0.052)	20.414	0.000	0.069
waist - AVI	0.001(0.001, 0.001)	21.669	0.000	0.069
waist - WWI	0.003(-0.001, 0.006)	1.331	0.183	0.069
hip - bodyfat	-0.052(-0.056, -0.048)	-25.441	0.000	0.069
hip - bmi	-0.056(-0.060, -0.052)	-27.744	0.000	0.069
hip - WHR	-0.073(-0.079, -0.067)	-23.406	0.000	0.069
hip - ABSI	-0.019(-0.025, -0.013)	-6.108	0.000	0.070
hip - AVI	-0.065(-0.069, -0.062)	-38.278	0.000	0.069
hip - WWI	-0.064(-0.069, -0.058)	-22.348	0.000	0.069
bodyfat - bmi	-0.004(-0.006, -0.001)	-2.708	0.007	0.069
bodyfat - WHR	-0.021(-0.026, -0.016)	-8.433	0.000	0.069
bodyfat - ABSI	0.034(-0.027, 0.040)	10.116	0.000	0.069
bodyfat - AVI	-0.013(-0.016, -0.010)	-7.335	0.000	0.069
bodyfat - WWI	-0.011(-0.017, -0.006)	-3.918	0.000	0.069
bmi - WHR	-0.017(-0.022, -0.012)	-7.011	0.000	0.069
bmi - ABSI	0.037(-0.030, 0.044)	10.564	0.000	0.069
bmi - AVI	-0.009(-0.013, -0.006)	-5.413	0.000	0.069
bmi - WWI	-0.008(-0.014, -0.002)	-2.593	0.010	0.069
WHR - ABSI	0.054(0.050, 0.058)	25.083	0.000	0.069
WHR - AVI	0.008(0.004, 0.011)	4.512	0.000	0.069
WHR – WWI	0.009(0.006, 0.013)	5.173	0.000	0.069
ABSI - AVI	-0.046(-0.051, -0.042)	-19.853	0.000	0.069
ABSI - WWI	-0.045(-0.047, -0.043)	-41.547	0.000	0.069
AVI - WWI	0.002(-0.022, 0.005)	0.784	0.433	0.069

Table S3. Comparisons of AUC for predicting hypertension among ORIs by the Delong test in female

comparator	AUC diff (95%CI)	*Z*	*P*	*Pa*
WHtR - waist	-0.020(-0.022, 0.004)	-25.916	0.000	0.067
WHtR - hip	-0.123(-0.128, -0.119)	-54.789	0.000	0.069
WHtR - bodyfat	-0.033(-0.036, -0.029)	-18.179	0.000	0.068
WHtR - bmi	-0.052(-0.055, -0.048)	-28.649	0.000	0.068
WHtR - WHR	-0.021(-0.024, -0.018)	-14.011	0.000	0.067
WHtR - ABSI	0.078(0.074, 0.082)	37.577	0.000	0.069
**WHtR - BRI**	0.000(0.000, 0.000)	0.554	**0.580**	0.067
WHtR - AVI	0.022(0.020, 0.024)	27.450	0.000	0.068
WHtR - WWI	0.028(0.025, 0.030)	20.914	0.000	0.068
BRI - waist	-0.020(-0.022, -0.019)	-25.916	0.000	0.067
BRI - hip	-0.123(-0.128, -0.119)	-54.789	0.000	0.069
BRI - bodyfat	-0.033(-0.036, -0.029)	-18.179	0.000	0.068
BRI - bmi	-0.052(-0.055, -0.048)	-28.649	0.000	0.068
BRI - WHR	-0.021(-0.024, -0.018)	-14.011	0.000	0.067
BRI - ABSI	-0.078(-0.082, -0.074)	-37.577	0.000	0.069
BRI - AVI	0.022(0.020, 0.024)	27.450	0.000	0.068
BRI - WWI	0.028(0.025, 0.030)	20.914	0.000	0.068
waist - hip	0.103(0.099, 0.107)	52.440	0.000	0.070
waist - bodyfat	0.013(0.009, 0.016)	7.169	0.000	0.068
waist – bmi	0.032(0.028, 0.035)	17.533	0.000	0.069
waist - WHR	0.001(-0.002, 0.004)	0.466	0.641	0.068
waist - ABSI	0.058(0.054, 0.062)	27.400	0.000	0.069
waist - AVI	0.002(0.002, 0.002)	24.398	0.000	0.068
waist - WWI	0.008(0.005, 0.011)	4.743	0.000	0.068
hip - bodyfat	-0.090(-0.095, -0.086)	-43.372	0.000	0.070
hip - bmi	-0.071(-0.075, -0.067)	-35.035	0.000	0.071
hip - WHR	-0.102(-0.109, -0.096)	-31.651	0.000	0.070

hip - ABSI	-0.045(-0.051, -0.039)	-13.890	0.000	0.071
hip - AVI	-0.101(-0.105, -0.097)	-53.044	0.000	0.070
hip - WWI	-0.095(-0.101, -0.090)	-32.500	0.000	0.070
bodyfat - bmi	0.019(0.017, 0.021)	16.918	0.000	0.069
bodyfat - WHR	-0.012(-0.017, -0.007)	-4.806	0.000	0.068
bodyfat - ABSI	0.045(0.039, 0.052)	13.931	0.000	0.069
bodyfat - AVI	-0.011(-0.014, -0.007)	-6.121	0.000	0.068
bodyfat - WWI	-0.005(-0.010, 0.001)	-1.770	0.077	0.069
bmi - WHR	-0.031(-0.036, -0.026)	-12.104	0.000	0.069
bmi - ABSI	0.026(0.019, 0.033)	7.537	0.000	0.070
bmi - AVI	-0.030(-0.033, -0.026)	-16.621	0.000	0.069
bmi - WWI	-0.024(-0.030, -0.018)	-8.204	0.000	0.069
WHR - ABSI	0.057(0.054, 0.061)	31.031	0.000	0.069
WHR - AVI	0.001(-0.002, 0.004)	0.740	0.459	0.068
WHR – WWI	0.007(0.004, 0.010)	4.654	0.000	0.068
ABSI - AVI	-0.056(-0.060, -0.052)	-26.084	0.000	0.069
ABSI - WWI	-0.050(-0.052, -0.048)	-51.679	0.000	0.069
AVI - WWI	0.006(0.003, 0.009)	3.479	0.001	0.068

Table S4. Sensitivity analysis for ROC curve analysis results for ORIs predicting hypertension in adults

ORIs	Overall AUC（95%CI）	*P*	Male AUC（95%CI）	*P*	Female AUC（95%CI）	*P*
WC	0.674 (0.671, 0.677)	<0.001	0.626 (0.622, 0.631)	<0.001	0.691 (0.686, 0.695)	<0.001
HC	0.594 (0.590, 0.579)	<0.001	0.566 (0.561, 0.657)	<0.001	0.596 (0.591, 0.601)	<0.001
BF%	0.578 (0.575, 0.582)	<0.001	0.617 (0.613, 0.622)	<0.001	0.681 (0.667, 0.686)	<0.001
BMI	0.650 (0.647, 0.653)	<0.001	0. 620 (0.615, 0.624)	<0.001	0.663 (0.658, 0.667)	<0.001
WHR	0.672 (0.669, 0.675)	<0.001	0.626 (0.622, 0.631)	<0.001	0.688 (0.683, 0.692)	<0.001
WHtR	0.679 (0.676, 0.682)	<0.001	0.644 (0.639, 0.648)	<0.001	0.707 (0.703, 0.712)	<0.001
ABSI	0.612 (0.609, 0.615)	<0.001	0.568 (0.564, 0.573)	<0.001	0.631 (0.626, 0.636)	<0.001
BRI	0.679 (0.676, 0.682)	<0.001	0.644 (0.639, 0.648)	<0.001	0.707 (0.703, 0.712)	<0.001
AVI	0.672 (0.669, 0.676)	<0.001	0.626 (0.621, 0.630)	<0.001	0.689 (0.685, 0.693)	<0.001
WWI	0.643 (0.640, 0.647)	<0.001	0.611 (0.607, 0.616)	<0.001	0.678 (0.674, 0.683)	<0.001

ORIs: Obesity-Related Indicators; WC, waist circumference; HC, hip circumference; BF%, body fat percentage; BMI, body mass index; WHR, waist-hip ratio; WHtR, waist- height ratio; ABSI, a body shape index; BRI, body roundness index; AVI, abdominal volume index; WWI, weight-adjusted-waist index; ROC, Receiver Operating Characteristic; AUC, Area Under the Curve

Table S5. Youden's Index and cut-off values of WHtR and BRI

Optimal ORIs	WHtR		BRI	
	Youden’s index	Cut-off values	Youden’s index	Cut-off values
Overall	0.267	0.51	0.267	3.52
Male	0.214	0.52	0.214	3.67
Female	0.310	0.51	0.310	3.53

Cut-off values were determined using the maximum Youden’s Index

Youden’s Index= Sensitivity + Specificity - 1

Table S6. Sensitivity analysis for comparisons of AUC for predicting hypertension between WHtR and ORIs by the Delong test in overall

**comparator**	**AUC diff (95%CI)**	***Z***	***P***	***Pa***
WHtR - waist	0.006 (0.004, 0.007)	7.690	0.000	0.056
WHtR – hip	0.086 (0.083, 0.089)	58.779	0.000	0.057
WHtR – bodyfat	0.101 (0.098, 0.104)	64.196	0.000	0.057
WHtR - bmi	0.030 (0.027, 0.032)	25.170	0.000	0.056
WHtR - WHR	0.007 (0.005, 0.009)	6.488	0.000	0.056
WHtR - ABSI	0.067 (0.064, 0.070)	46.472	0.000	0.056
**WHtR - BRI**	0.000 (0.000, 0.000)	-0.344	**0.731**	0.056
WHtR – AVI	0.007 (0.005, 0.008)	9.317	0.000	0.056
WHtR - WWI	0.036 (0.034, 0.038)	37.741	0.000	0.056

Table S7. Sensitivity analysis for comparisons of AUC for predicting hypertension between WHtR and ORIs by the Delong test in male

**comparator**	**AUC diff (95%CI)**	***Z***	***P***	***Pa***
WHtR - waist	0.017(0.016, 0.019)	19.123	0.000	0.068
WHtR – hip	0.078(0.074, 0.082)	37.188	0.000	0.068
WHtR – bodyfat	0.027(0.023, 0.030)	13.998	0.000	0.068
WHtR - bmi	0.024(0.021, 0.027)	13.995	0.000	0.068
WHtR - WHR	0.018(0.015, 0.021)	11.355	0.000	0.068
WHtR - ABSI	0.076(0.071, 0.080)	34.079	0.000	0.068
**WHtR - BRI**	0.000(0.000, 0.000)	1.157	**0.247**	0.067
WHtR – AVI	0.018(0.016, 0.020)	19.775	0.000	0.068
WHtR - WWI	0.032(0.030, 0.035)	22.041	0.000	0.068

Table S8. Sensitivity analysis for comparisons of AUC for predicting hypertension between WHtR and ORIs by the Delong test in female

comparator	AUC diff (95%CI)	*Z*	*P*	*Pa*
WHtR - waist	0.017 (0.015, 0.018)	22.939	0.000	0.068
WHtR – hip	0.067 (0.107, 0.115)	52.909	0.000	0.067
WHtR – bodyfat	0.026 (0.023, 0.029)	15.452	0.000	0.066
WHtR - bmi	0.045 (0.041, 0.048)	26.650	0.000	0.067
WHtR - WHR	0.020 (0.017, 0.022)	14.243	0.000	0.066
WHtR - ABSI	0.067 (0.073, 0.080)	39.188	0.000	0.067
**WHtR - BRI**	0.000 (0.000, 0.000)	-0.101	**0.920**	0.066
WHtR – AVI	0.018 (0.017, 0.020)	24.469	0.000	0.066
WHtR - WWI	0.029 (0.027, 0.031)	23.361	0.000	0.067

Table S9. Sensitivity analysis for comparisons of AUC for predicting hypertension between BRI and ORIs by the Delong test in overall.

comparator	AUC diff (95%CI)	*Z*	*P*	*Pa*
BRI - waist	0.006 (0.004, 0.007)	7.690	0.000	0.056
BRI – hip	0.086 (0.083, 0.089)	58.779	0.000	0.056
BRI – bodyfat	0.101 (0.098, 0.104)	64.196	0.000	0.056
BRI - bmi	0.030 (0.027, 0.032)	25.170	0.000	0.056
BRI - WHR	0.007 (0.005, 0.009)	6.488	0.000	0.056
**BRI - WHtR**	0.000 (0.000, 0.000)	0.344	**0.731**	0.056
BRI - ABSI	0.067 (0.064, 0.070)	46.472	0.000	0.056
BRI– AVI	0.007 (0.005, 0.008)	9.317	0.000	0.056
BRI - WWI	0.036 (0.034, 0.038)	37.741	0.000	0.056

Table S10. Sensitivity analysis for comparisons of AUC for predicting hypertension between BRI and ORIs by the Delong test in male.

**comparator**	**AUC diff (95%CI)**	***Z***	***P***	***Pa***
BRI - waist	0.017 (0.016, 0.019)	19.123	0.000	0.068
BRI – hip	0.078 (0.074, 0.082)	37.188	0.000	0.068
BRI – bodyfat	0.027 (0.023, 0.030)	13.998	0.000	0.068
BRI - bmi	0.024 (0.021, 0.027)	13.994	0.000	0.068
BRI - WHR	0.018 (0.017, 0.022)	14.243	0.000	0.068
**BRI - WHtR**	0.000 (0.000, 0.000)	-1.157	**0.247**	0.067
BRI - ABSI	0.076 (0.071, 0.080)	34.079	0.000	0.068
BRI– AVI	0.018 (0.016, 0.020)	19.775	0.000	0.068
BRI - WWI	0.032 (0.030, 0.035)	22.041	0.000	0.068

Table S11. Sensitivity analysis for comparisons of AUC for predicting hypertension between BRI and ORIs by the Delong test in female.

**comparator**	**AUC diff (95%CI)**	***Z***	***P***	***Pa***
BRI - waist	0.017 (0.015, 0.018)	22.939	0.000	0.068
BRI – hip	0.111 (0.107, 0.115)	52.909	0.000	0.067
BRI – bodyfat	0.026 (0.023, 0.029)	15.452	0.000	0.066
BRI - bmi	0.045 (0.041, 0.048)	26.650	0.000	0.067
BRI - WHR	0.020 (0.017, 0.022)	14.243	0.000	0.066
**BRI - WHtR**	0.000 (0.000, 0.000)	0.101	0.920	0.066
BRI - ABSI	0.079 (0.073, 0.080)	39.188	0.000	0.067
BRI– AVI	0.018 (0.017, 0.020)	24.469	0.000	0.066
BRI - WWI	0.029 (0.027, 0.031)	23.361	0.000	0.067

Table S12. Sensitivity analysis for the association of WHtR, BRI and hypertension in multivariate logistic regression models

Optimal ORIs	Overall<br>*OR* (95%*CI*)		Male<br>*OR* (95%*CI*)		Female<br>*OR* (95%*CI*)
WHtR					
WHtR z-score					
	1.53(1.50,1.55)		1.53(1.50, 1.56)		1.45(1.42,1.48)
Dichotomous category of WHtR					
＜0.51	Ref (1.00)	＜0.52	Ref (1.00)	＜3.51	Ref (1.00)
≥0.51	1.94(1.89, 1.99)	≥0.52	1.88(1.81, 1.95)	≥3.51	1.83(1.76, 1.91)
BRI					
BRI z-score					
	1.52(1.50,1.54)		1.53(1.50,1.56)		1.44(1.41, 1.47)
Dichotomous category of BRI					
＜3.52	Ref (1.00)	＜3.67	Ref (1.00)	＜3.53	Ref (1.00)
≥3.52	1.95(1.90, 2.00)	≥3.67	1.89(1.82, 1.96)	≥3.53	1.84(1.77, 1.91)

2.1 Supplementary Figures

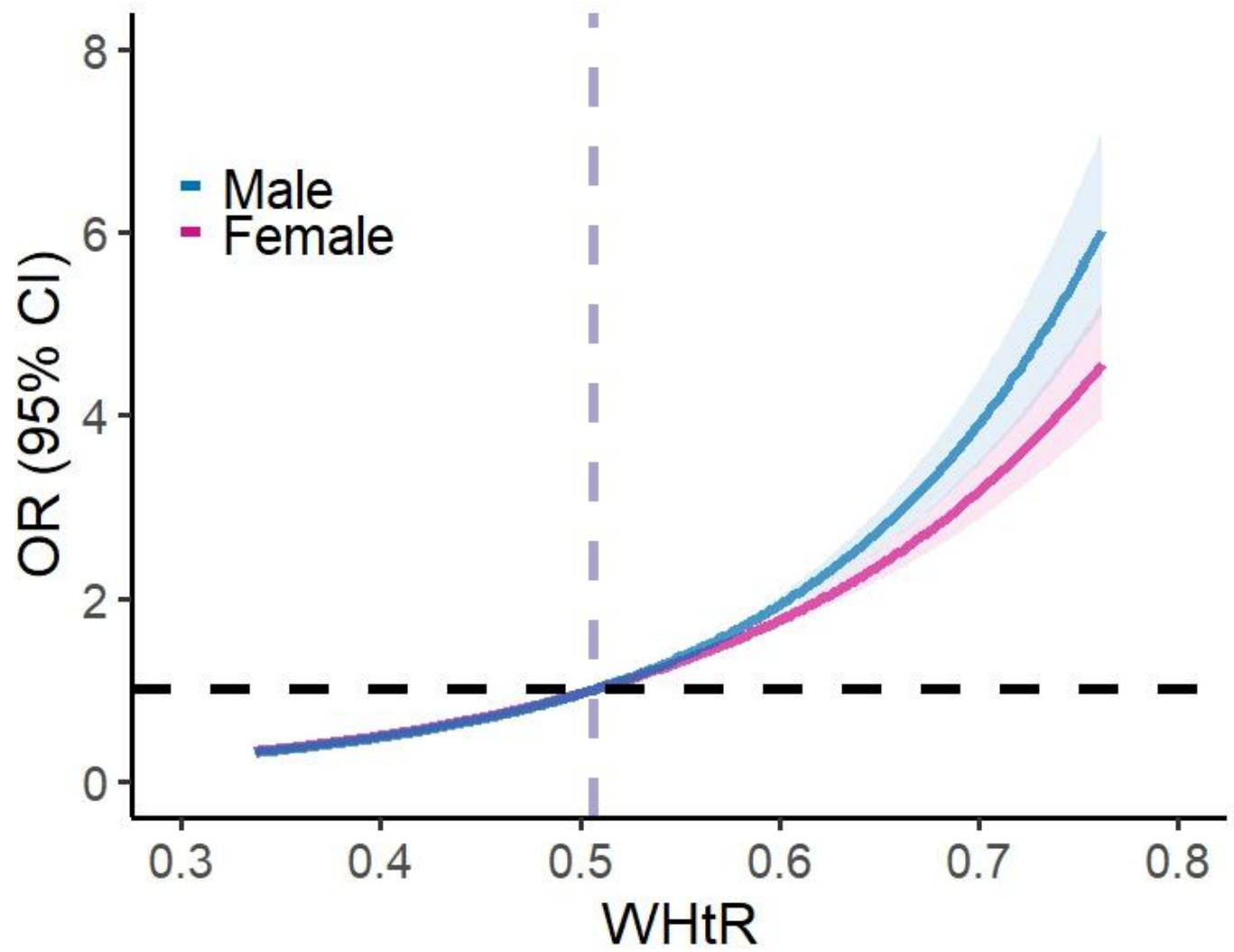

Supplementary Figure 1. Sensitivity analysis for dose-response relationship between WHtR and hypertension

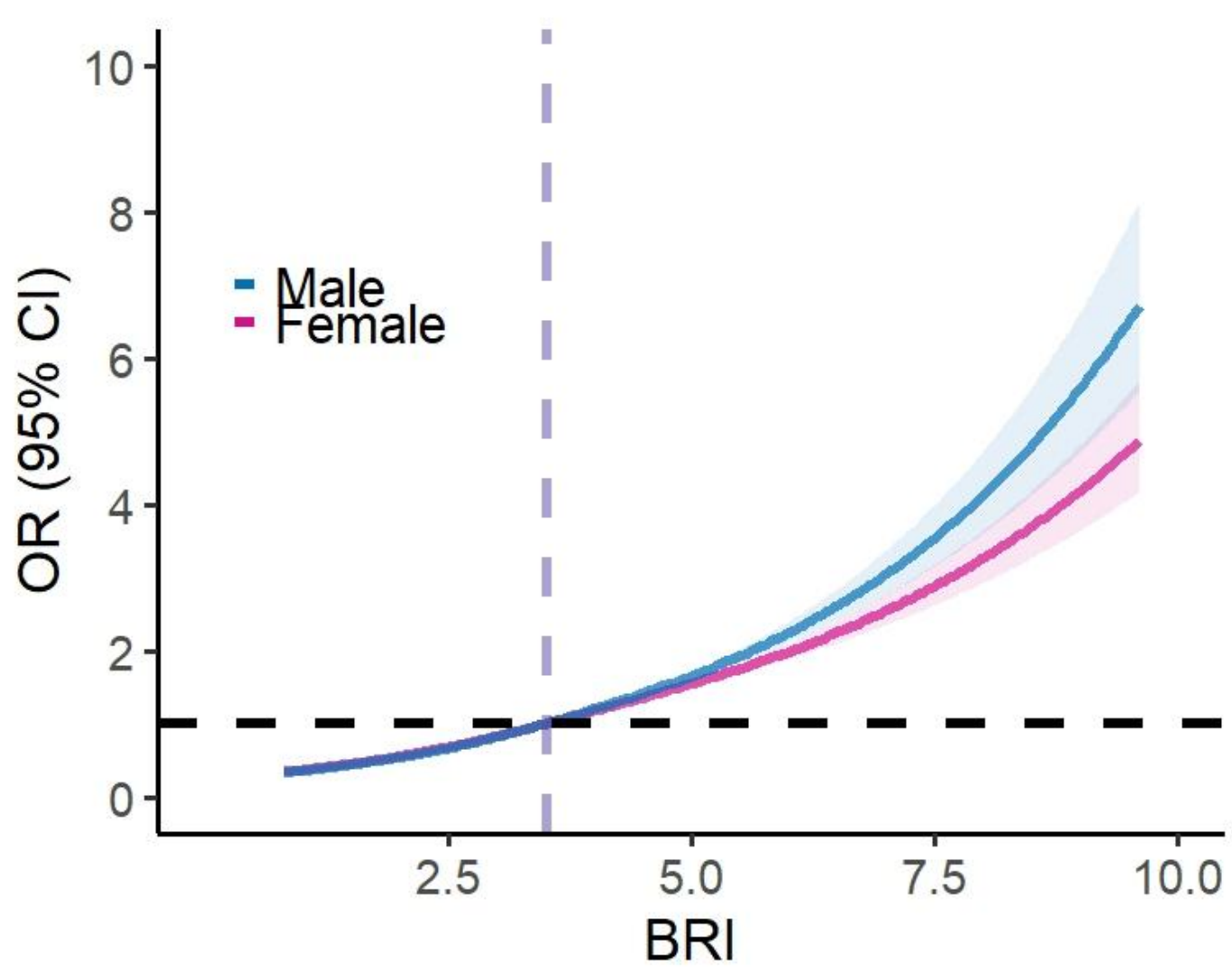

Supplementary Figure 2. Sensitivity analysis for dose-response relationship between BRI and hypertension
